# Expression of HOXC8 is inversely related to the progression and metastasis of pancreatic ductal adenocarcinoma

**DOI:** 10.1038/bjc.2011.217

**Published:** 2011-06-28

**Authors:** H Adwan, M Zhivkova-Galunska, R Georges, E Eyol, J Kleeff, N A Giese, H Friess, F Bergmann, M R Berger

**Affiliations:** 1 Toxicology and Chemotherapy Unit, German Cancer Research Center, Deutsches Krebsforschungszentrum Heidelberg, Im Neuenheimer Feld 581, Heidelberg D-69120, Germany; 2 Department of General Surgery, Technical University of Munich, Munich, Germany; 3 Department of General Surgery, University of Heidelberg, Heidelberg, Germany; 4 Institute of Pathology, University of Heidelberg, Heidelberg, Germany

**Keywords:** HOXC8, pancreatic ductal adenocarcinoma, liver metastasis, osteopontin, osteonectin

## Abstract

**Background:**

The transcription factor HOXC8 regulates many genes involved in tumour progression. This study was to investigate the role of HOXC8 in pancreatic ductal adenocarcinoma (PDAC) growth and metastasis.

**Methods:**

The Hoxc8 expression was determined in 15 PDAC cell lines and human specimens by RT–polymerase chain reaction and/or immunohistochemistry. The effects of HOXC8 silencing by RNA interference were investigated by functional tests.

**Results:**

The Hoxc8 mRNA expression in PDAC cell lines was negatively related to their growth *in vivo*. Except for Suit2-007 cells, only those with low Hoxc8 mRNA expression grew in nude rats. Successful down-regulation of HOXC8 expression caused increased proliferation, migration (*P*⩽0.05) and colony formation (*P*⩽0.05) in Suit2-007, Panc-1 and MIA PaCa-2 PDAC cells, respectively. The Hoxc8 mRNA levels in diseased human pancreas tissues were significantly increased over normal in PDAC and autoimmune chronic pancreatitis specimens (*P*<0.01, respectively), but negatively related to tumour stage (*P*=0.09). In primary and metastatic tumour samples, immunohistochemical staining for HOXC8 was stronger in surrounding than in neoplastic tissues. Furthermore, grading of primary carcinomas was negatively associated with HOXC8 staining (*P*=0.03). Liver metastases showed the lowest HOXC8 expression of all neoplastic lesions.

**Conclusion:**

These data indicate that HOXC8 expression is inversely related to PDAC progression and metastasis.

Pancreatic ductal adenocarcinoma (PDAC) is the third-most frequent neoplasia of the GI-tract and the fifth leading cause of cancer-related mortality in industrialised countries. With an overall 5-year survival rate of 1–3% and a median survival time of 5–6 months after diagnosis, PDAC is considered to be one of the most aggressive cancers (Jemal *et al*, 2005). Lack of early symptoms and reliable screening tests for early detection are main causes for the fact that over 80% of patients with PDAC are already inoperable at the time of diagnosis (Li *et al*, 2004).

Since cancer is a genetic disease, an improved understanding of gene-expression alteration in PDAC and its distant metastasis is a reasonable way of identifying pathways, which are causal for tumour progression and metastasis. In addition, new markers for early diagnosis as well as potential targets for therapeutic intervention can be expected from that approach (Li *et al*, 2004). Following these lines, the extra-cellular matrix proteins osteonectin (ON) and osteopontin (OPN) have been found up-regulated by gene array in PDAC. We could show that ON is markedly over-expressed in pancreatic cancer and chronic pancreatitis (CP) (Guweidhi *et al*, 2005). Osteonectin or secreted protein acidic and rich in cysteine is a calcium-binding, anti-adhesive and bone-specific glycoprotein with high affinity to collagen and hydroxyapatite. Its expression is associated with processes such as morphogenesis, angiogenesis, cell differentiation, proliferation and migration, as well as wound healing (Brekken and Sage, 2001). In addition, the expression of ON has been related to cancer progression, an over-expression of ON was found in breast, colon and prostate cancers (Porte *et al*, 1995; Graham *et al*, 1997; Thomas *et al*, 2000). Remarkably, other studies assigned ON a tumour suppressor role, where its de-regulation seems to be related to tumour progression and/or poor prognosis (Podhajcer *et al*, 2008).

We also could show that OPN is markedly up-regulated in pancreatic cancer (Kolb *et al*, 2005). The secreted, adhesive non-collagenous phosphorylated calcium-binding glycoprotein OPN (Fisher *et al*, 2001) is associated with the progression of colon, papillary thyroid, lung, breast and prostate cancers, with its elevated expression being linked to a poor prognosis (Brown *et al*, 1994; Fedarko *et al*, 2001). Osteopontin has been found up-regulated by various genes, for example TGF-*β* and HOXC8 (Roy and Sen, 2005; Shen *et al*, 2007).

The HOXC8 belongs to the homeobox class I family. Like other members of this family, HOXC8 regulates anterior–posterior patterning and is jointly responsible for skeletal and neural development (Thickett and Morgan, 2002; Juan *et al*, 2006). In the mouse, HOXC8 is expressed in the neural tube and somitic mesoderm as well as in the prospective thorax (Belting *et al*, 1998) and is crucial for mouse skeletal and forelimb development (Le *et al*, 1992). The tissue-specific over-expression of HOXC8 inhibits the maturation and stimulates the proliferation of chondrocytes, resulting in cartilage defects (Yueh *et al*, 1998). The HOXC8 is also expressed in haematopoietic organs, fetal liver and adult bone marrow (Shimamoto *et al*, 1999). Hox-binding elements (ATTA) are involved in promoters of osteoblast differentiation and osteogenesis marker genes, such as osteoprotegerin, BMP-4 and ON (Wan *et al*, 2001; Roy and Sen, 2005). Furthermore, HOXC8 seems to have an essential role in cancer development and progression. In several cancer types, HOXC8 is down-regulated, such as in oesophageal and prostate cancers (Miller *et al*, 2003; Chen *et al*, 2005). In human prostate cancer, HOXC8 is both down-regulated as well as up-regulated in association with loss of tumour differentiation. Its complex role seems to promote invasiveness, while inhibiting androgen receptor-mediated gene induction at androgen response element-regulated genes associated with differentiated function of the prostate (Axlund *et al*, 2010). This suggests that HOXC8 may have a role in both the acquisition of the invasive and metastatic phenotype of this malignancy as well as in inhibiting androgen responsive prostate cancer cells (Waltregny *et al*, 2002; Miller *et al*, 2003).

The aim of this study was to investigate the HOXC8 expression in PDAC and its liver metastasis in comparison with healthy and inflamed pancreatic tissues. In addition, the effect of Hoxc8 knockdown by RNA interference was investigated on cell proliferation, migration and colony formation. Finally, the relationship of HOXC8 to OPN- or ON-expression levels was to be determined in pancreatic cancer cell lines and related to their growth *in vivo*.

## Materials and methods

### Cell culture

One rat (ASML) and 14 human pancreatic carcinoma cell lines were used for detecting the expression of HOXC8 and for transplantation into animals (see Supplementary Table). All cells were kept in log-phase, and passaged 2–4 times per week depending on their growth rate, and were maintained in an incubator at 37°C in humidified air with 5% CO_2_.

### Patients and tissue collection

Human pancreatic cancer tissue samples were obtained from 47 patients (25 women, 22 men, median age 64 years (range, 39–80 years)) and CP tissue samples from 37 patients (11 women, 26 men, median age 54 years (range, 25–73 years)) who underwent pancreatic resection at the University Hospitals of Bern (Switzerland) and Heidelberg (Germany). Normal human pancreatic tissue samples were obtained through an organ donor programme from 10 previously healthy individuals (four women, six men, 15–69 years median age 45 years). Freshly removed tissue samples were immediately fixed in paraformaldehyde solution for 12–24 h and paraffin embedded for immunohistochemical analysis. Concomitantly, tissue samples for RNA extraction were immediately snap frozen in liquid nitrogen in the operating room and maintained at −80°C until analysis.

#### Immunohistochemistry

Paraffin-embedded tissue sections from 37 PDAC and 12 liver metastasis specimens were analysed as described in Supplementary methods S1.

#### Western blot analysis

Western blotting was performed as described previously (Zhivkova-Galunska *et al*, 2010). Primary antibodies for HOXC8, OPN, ON and ERK2 as well as their corresponding secondary antibodies are given in Supplementary methods S2.

### Real-time light cycler quantitative polymerase chain reaction

All reagents and equipment for mRNA/cDNA preparation were purchased from Roche Applied Science (Mannheim, Germany). mRNA was prepared by automated isolation using MagNA Pure LC instrument and isolation kits I (for cells) and II (for tissue samples). cDNA was prepared using the first Strand cDNA Synthesis kit for RT–polymerase chain reaction (PCR) according to the manufacturer's instructions. Real-time PCR was performed with the Light Cycler Fast Start DNA SYBR Green kit as described previously (Guo *et al*, 2004) (all primers used are shown in Table 1). The number of specific (Hoxc8) transcripts was normalised to housekeeping genes (cyclophilin-B and hypoxanthine guanine phosphoribosyltransferase). All primers were obtained from Search-LC (Heidelberg, Germany).

### Hoxc8 siRNA transfection of human pancreatic cancer cells

Cells were plated overnight at a density of 250 000 cells per well in six-well plates. A total of 100 *μ*l transfection solution containing 6 ng (final concentration 100–200 nM) of three different Hoxc8 siRNAs (siRNA oligomers used are shown in Table 1) or negative control siRNA (Invitrogen, Karlsruhe, Germany) and 15 *μ*l transfection reagent (Invitrogen) were added to 1.9 ml medium per well. After 12 h, cells were trypsinised and used for subsequent protein or mRNA extraction, for immunoblot analysis or the *in vitro* assays mentioned above.

### MTT assay

To assess the effect of siRNA transfection on the proliferation of Suit2-007, Panc-1 or MIA PaCa-2 cells, the 3-(4,5-dimethylthiazol-2-yl)-2,5-diphenyltetrazolium bromide (MTT) assay was used (Supplementary methods S3).

### Clonogenicity assay

For determining the response of Suit2-007, Panc-1 and MIA PaCa-2 colony formation after exposure to siRNA oligonucleotides directed against Hoxc8 mRNA, the procedure previously detailed (Adwan *et al*, 2004) was performed. Clusters of 30 cells were counted as colony, whereas clusters of ⩾60 cells were considered as large colony.

### *In vitro* cell migration model

This assay was performed to investigate the effect of HOXC8 down-regulation on the migration of Suit2-007, Panc-1 and MIA PaCa-2 cells. The bottom layer in 24-well plates consisted of 50 *μ*l FCS, which was gently over-layered with 200 *μ*l semi-liquid RPMI medium (containing 0.4% methylcellulose and 20% FCS) resulting in the chemotaxis mixture. A period of 24 h was needed to build the chemotaxis gradient. Then, 1 × 10^4^ Suit2-007, Panc-1 or MIA PaCa-2 cells were seeded on 8 *μ*m pore size polycarbonate membranes (Millicell; Millipore, Schwalbach, Germany), which were transferred onto the prepared wells. The next day, the cells were exposed to siRNA directed against HOXC8 for 1–3 days, and then plated onto the polycarbonate inserts. The inserts were removed from the bottom layer after 24 h of co-cultivation and transferred onto a fresh well of the same plate with chemotaxis gradient. Cells migrating through the pores were then counted daily for 4 days by fluorescence microscopy.

### Animals and husbandry

Nude rats (RNU strain) were obtained from Harlan or Charles River (Harlan comp, Borchen, Germany; Charles River, Sulzfeld, Germany) at an age of 6–8 weeks. They were housed under specific pathogen-free conditions in a mini-barrier system of the central animal facility. Autoclaved feed and water was given *ad libitum* to the animals that were maintained under controlled conditions (21±2°C room temperature, 60% humidity and 12 h light–dark rhythm).

### Pancreatic liver lesions *in vivo*

To induce liver lesions, approximately 2 × 10^7^ cells (15 different cell lines used) were injected either intraportally via a mesocolic vein or under the liver capsule of a nude rat. In the case of a positive outcome, some tumour growth could be visually detected during re-laparotomy in the liver after a period of 7–14 days. The animals were euthanised after 4 weeks and examined a second time for the presence of liver metastases.

### Statistics

The results of multiple measurements were given as mean with corresponding standard deviation. The effect of siRNA on cell proliferation, migration and colony formation was described as treated/control × 100 (T/C%). Differences between treated and control groups were assessed by the Kruskal–Wallis test, a non-parametric rank sum test. The same test was used to compare the mRNA-expression levels between normal and diseased pancreatic tissues. The χ^2^-test was used to examine for independent occurrence of investigated parameters in cell lines (expression of genes and growth *in vivo*). A *P*-level ⩽0.05 was considered significant.

## Results

### Expression and localisation of HOXC8 in pancreatic tissues

**Figure 1:**
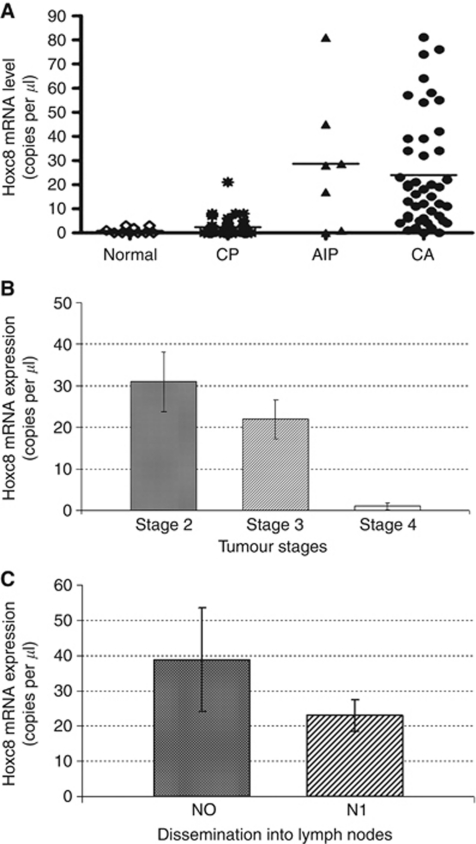
Hoxc8 mRNA expression in diseased and normal pancreatic tissues. (**A**) Expression levels in 10 normal pancreatic tissues, 37 chronic pancreatitis (CP) tissues, 6 autoimmune pancreatitis (AIP) tissues and 47 pancreatic cancer adenocarcinoma (PDAC) tissues. Horizontal lines represent the respective median. (**B**) Expression level in relation to tumour grading (mean with error bars). (**C**) Expression level in relation to nodal status (mean with error bars).

Quantitative RT–PCR was used to compare the *in vivo* expression profile of Hoxc8 in normal and diseased pancreatic tissues. The expression of HOXC8 mRNA in all 10 donor samples was extremely low: in 6 of 10 samples even under the detection limit (less than one copy per *μ*l), while in the other samples only 1–4 mRNA copies were detected. Compared with these levels, there was a slight but not significant increase in Hoxc8 mRNA expression in the 37 samples of patients with CP (2.4-fold, *P*=0.09; Figure 1A) with 50% of all samples being below the detection limit. In contrast, this analysis revealed a 24-fold increase in mean Hoxc8 mRNA level in 47 PDAC samples and a corresponding 28-fold increase in 6 autoimmune CP specimen as compared with normal pancreatic tissue (*P*<0.01, respectively). Remarkably, an inverse relation was found between tumour grade and expression level as shown in Figure 1B. Similarly, tumour samples from patients that were characterised as nodal positive (N1) harboured significantly less Hoxc8 mRNA copies than samples from N0 patients (Figure 1C).

To determine the cellular source and localisation of HOXC8 in pancreatic tissues, 15 CP as well as 31 primary and 12 metastatic PDAC tissues were probed for immunoreactivity. This analysis showed that staining for HOXC8 was always stronger in the surrounding than in the neoplastic tissues. Pancreatitis samples showed more intensive immunoreactivity than PDAC tissues.

From a total of 31 PDAC samples, 21 showed some immunoreactivity: only 4 samples stained clearly positive. The remaining 17 varied in their staining intensity from moderate, weakly positive (*n*=8) to faintly, focal positive (*n*=9). No HOXC8 immunoreactivity was detected in 10 samples. Grading of primary carcinomas was negatively associated with the extent and intensity of HOXC8 staining (*P*=0.03).

Furthermore, weak-to-faint HOXC8 immunoreactivity was observed in the 9 of 12 PDAC liver metastasis specimens and three were completely negative.

**Figure 2:**
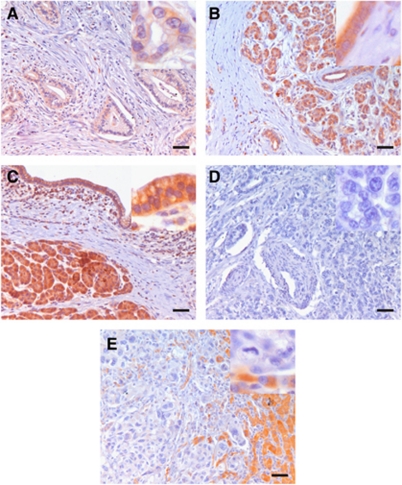
Immunohistochemistry of HOXC8 expression in diseased pancreatic tissue samples shows that HOXC8 expression (coloured in red brown) was always stronger in surrounding than in neoplastic tissues. Pancreatitis samples showed more intensive immune-reactivity than PDAC tissues: (**A**) autoimmune pancreatitis, (**B**) CP, (**C** and **D**) PDAC and (**E**) liver metastases of PDAC. The scale bars indicate a distance of 100 *μ*m, the upper right corner shows a five-fold higher magnification, respectively.

In contrast, in AIP and CP tissue samples, strong, diffuse HOXC8 immunoreactivity was observed in the tubular complexes, degenerating acinar cells and islands as well as in the extra-cellular matrix and nerves (Figures 2A and B).

### The functional role of HOXC8 in pancreatic cancer cell lines

#### *In vitro*

To examine the functional role of HOXC8 in pancreatic cancer, 15 pancreatic cancer cell lines were analysed for the expression of Hoxc8 mRNA by quantitative RT–PCR (primers are shown in Table 1). The levels of Hoxc8 mRNA were high in DANG, Panc-1 and Suit2-007 pancreatic cancer cells, relatively high to moderate in A8 18–4, MIA PaCa-2, Capan 1, Patu 390, as well as SU 8686 cells, and low to very low in CFPAC, Aspc-1, Panc-89, Colo-357, ASML, S2013 and BxPc-3 cells.

Although there was no significant correlation between the expression levels of Hoxc8 mRNA and differentiation or basal growth characteristics of the cell lines *in vitro*, there was a noteworthy relationship regarding their growth behaviour *in vivo* (see below).

#### *In vivo*

*In vivo*, there was a significant inverse relation in 14 out of 15 cell lines (93% *P*=0.002) between the cells’ ability to grow in the liver of nude rats and their Hoxc8 mRNA expression (Table 2). All seven cell lines with low to extremely low Hoxc8 mRNA expression were able to extravasate, to escape the primary immune response mediated by Kupffer cells, as well as to form lesions in the liver. In addition, no cell line with moderate (SU 8686) or relatively high Hoxc8 mRNA expression (8 MIA PaCa-2, Capan 1 and PATU 390) developed lesions in the liver. The only exception of this finding was a cell line (Suit2-007) with high Hoxc8 mRNA level, which was able to form liver lesions (Table 2).

### Relationship of HOXC8 to OPN and ON

**Figure 3:**
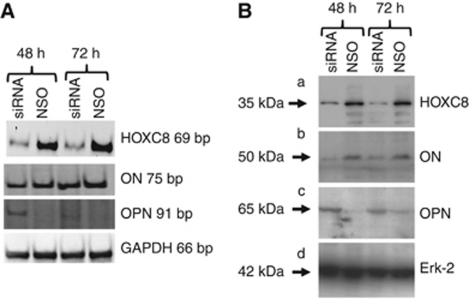
Detection of HOXC8, osteonectin (ON) and osteopontin (OPN) following treatment with siRNA directed against HOXC8: (**A**) RT–PCR in MIA PaCa-2 cells with GAPDH as house keeping gene and (**B**) western blot in Suit2-007 cells with Erk-2 as house keeping gene.

There was a significant inverse relationship between Hoxc8 and OPN mRNA expression in 13 of 15 cell lines (86.7% *P*=0.005), as well as a significant direct relationship between Hoxc8 and ON mRNA expression in 11 of 15 cell lines (73% *P*=0.05) (Table 2). In addition, inhibition of Hoxc8 with siRNA caused reduced expression of ON, but stimulation of OPN, as shown in Figure 3. Exposure to siRNA species directed against Hoxc8 inhibited its expression to 15%, respectively, as shown by RT–PCR and western blot (Figures 3A and B).

To further investigate a possible interdependence of Hoxc8 with OPN and ON, these two genes were investigated in parallel. Osteopontin expression was increased at mRNA (six-fold) and protein (three-fold) levels. In contrast, ON expression was down-regulated by 60% at mRNA and 80% at protein levels.

### Effect of HOXC8 on the growth of pancreatic cancer cells *in vitro*

**Figure 4:**
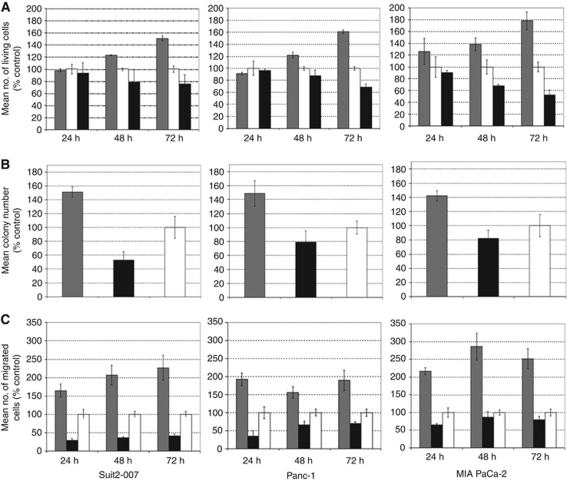
(**A**) Determination of cell proliferation by MMT assay after transfection of Suit2-007, Panc-1 and MIA PaCa-2 cells with siRNA against HOXC8 (grey) or NSO (black), as well as untreated control (white). The columns indicate the mean number of living cells in per cent of untreated control±s.d. (**B**) Effect of RNA interference-mediated HOXC8 knockdown on colony formation. Bars denote the number of colonies following exposure to siRNA directed against HOXC8 mRNA (grey columns), nonsense siRNA (black columns) or solvent (white columns) in Suit2-007, Panc-1 and MIA PaCa-2 pancreatic cancer cells. The columns indicate the mean colony number in per cent of untreated control±s.d. (**C**) Effect of RNA interference-mediated HOXC8 knockdown on the spontaneous trans-well migration. Bars (grey: HOXC8; black: NSO; white: untreated control) denote the number of migrating Suit2-007, Panc-1 and MIA PaCa-2 pancreatic cancer cells on days 1–3 after treatment. The columns indicate the mean number of migrated cell in per cent of untreated control±s.d.

For further investigations, three cell lines with high level of Hoxc8 mRNA (Suit2-007, Panc-1 and MIA PaCa-2 cells) were selected, one of which with the ability to form liver lesions (Suit2-007). A successful down-regulation of Hoxc8 by siRNA (Figure 4A) increased proliferation *in vitro* by 51%, 60% and 78% compared with untreated controls for Suit2-007, Panc-1 and MIA PaCa-2 cells, respectively (Figure 4A).

### Colony formation

Colony formation was used for studying the effect of HOXC8 down-regulation on the ability of Suit2-007, Panc-1 and MIA PaCa-2 pancreatic cancer cells to form clusters of >30 cells. In fact, siRNA-treated cells formed 2.9-fold (Suit2-007), 1.9-fold (Panc-1) and 1.7-fold (MIA PaCa-2) more colonies than NSO-treated cells (*P*⩽0.05; see Figure 4B). As untreated cells formed more colonies than the NSO-treated cells, the difference of the former experimental group to siRNA-treated cells was only 1.5-fold.

### Migration

Migration was used to further characterise the influence of HOXC8 on cellular properties related to metastasis. After knockdown of Hoxc8 by RNA interference in three cell lines (Suit2-007, Panc-1 and MIA PaCa-2), a significant increase in migration was observed (*P*⩽0.05). Compared with the respective NSO treatment, the number of migrating cells increased more than five-fold in Suit2-007, more than three-fold in Panc-1 as well as in MIA PaCa-2 cells within the observation period (24 h, left; 48 h, middle; 72 h, right; Figure 4C).

## Discussion

The family of *Hox* genes encodes transcription factors that regulate and coordinate the expression of several genes involved in embryonic development, differentiation and malignant transformation (Cillo *et al*, 2001). In humans, 39 class I Hox genes have been identified and grouped into four clusters (A, B, C and D) (Cillo *et al*, 2001). It has been shown that Hox genes are expressed in endothelial cells and are involved in the acquisition of the angiogenic phenotype. A relation between de-regulated Hox gene expression and malignant transformation has been reported by many independent studies, not only for leukaemias but also in solid tumours such as breast, cervical, ovarian, prostate and colorectal cancers as well as in melanoma and squamous cell carcinoma. Originally, up-regulation was thought to promote malignancy, but, more recently, both oncogenic and tumour suppressor functions have been attributed to *Hox* genes (Abate-Shen, 2002; Hung *et al*, 2003; Miller *et al*, 2003).

This study sought to identify the function of HOXC8 in PDAC and to determine whether there is an interaction between HOXC8 and the non-collagenous proteins OPN and ON. In general, the role of HOXC8 in cancer development has not yet been clearly defined.

For deciphering the function of HOXC8 in PDAC, we initially analysed the expression of Hoxc8 mRNA and protein in diseased and healthy pancreatic tissues samples, respectively. The Hoxc8 showed only basal mRNA expression in adult pancreatic tissue (<5 copies per *μ*l), but was markedly over-expressed in PDAC and AIP tissues. In comparison, its expression in CP was not significantly increased. This contradicts to the increased presence of this protein in CP tissues, as assessed by immunohistochemistry. We speculate that CP tissues used for mRNA extraction may have undergone damage *in vivo* because of auto-digestion. This type of error can be excluded for patho-histologic examinations because the pathologist will base his assessment on intact tissues. Remarkably, an inverse relation was found between tumour grade and expression level of Hoxc8 mRNA, indicating that the loss in HOCX8 mRNA expression is related to tumour progression. In line with this, the expression of HOXC8 protein in PDAC tissues was inversely associated with both tumour grade and liver metastasis. This pictures a factor, which shows low expression in normal tissue, is up-regulated in premalignant tissue and pancreatitis, but low again in metastatic tissue, thus possibly suggesting a temporal role for HOXC8 expression in tumour progression. However, the observation that the staining intensity of HOXC8 was higher in the surrounding ECM, including broblasts and endothelial cells, as well as in tissue adjacent to a metastasis than in the tumour cells themselves indicates that HOXC8 may rather have a defensive role against malignant PDAC cells.

This assumption is in line with our subsequent functional analysis showing increased proliferation, colony formation and migration of tumour cells after Hoxc8-mRNA down-regulation. Another support results from the *in vivo* part of this study. The expression of HOXC8 in 14 of 15 human pancreatic cancer cell lines was inversely related to their ability to grow in the liver of nude rats. Further support for the assumption that HOXC8 has a defensive role against tumour cell growth is derived from the fact that this transcription factor regulates proteins, such as OPN and ON, which are involved in cancer progression. It has been described by others and us that HOXC8 knockdown is associated with increased expression of OPN, which in turn stimulates the proliferation of cancer cells via two different pathways. First, OPN can act as a growth factor itself or can inhibit the onset of apoptosis. Furthermore, we have also shown that the inhibition of ON can stimulate the growth of cancer cells (Adwan *et al*, 2004; Lei *et al*, 2005; Zhivkova-Galunska *et al*, 2010).

Physiologically, OPN and ON are extra-cellular calcium-binding glycoproteins, which participate in the bone mineralisation via hydroxyapatite binding. In addition, they have functions as signalling molecules, either as cytokine (OPN) or for wound healing and angiogenesis (ON). Pathophysiologically, they share an increased expression in a series of malignant tumours, especially in those with skeletal involvement. These functions have been recently reviewed (Brown *et al*, 1994; Lane and Sage, 1994; Porter *et al*, 1995; Giachelli and Steitz, 2000; Brekken and Sage, 2001; Agrawal *et al*, 2002; Zhivkova-Galunska *et al*, 2010).

With regard to OPN, there was a significantly inverse relation between the mRNA-expression levels of Hoxc8 and OPN in 12 of 14 human pancreatic cancer cell lines investigated. Osteopontin has been associated with tumour progression in various types of cancer such as breast, colon, prostate and pancreatic carcinomas. Our functional analysis in Panc-1 and MIA PaCa-2 cells revealed an up-regulation of OPN mRNA in response to RNA interference-mediated down-regulation of Hoxc8.

In partial contrast, Suit2-007 cells expressing a genuinely high OPN level did not further increase OPN transcription upon HOXC8 silencing. Nevertheless, the functional parameters (proliferation, migration and colony formation) did increase in response to HOXC8 down-regulation. As the tumour cells grew in the liver of nude rats, we assume that HOXC8 lost its function for regulating OPN in this cell line, but not for those factors that are responsible for the aforementioned functional properties.

The direct relationship between HOXC8 and OPN was less significant than the inverse association between the former protein and ON (*P*=0.002 *vs P*=0.05). Nevertheless, knockdown of Hoxc8 in Suit2-007 cells by RNA interference was followed by down-regulation of ON, indicating that this order of events is still intact, other than that for OPN. Interestingly, the inverse relationship between HOXC8 and ON was less prominent at the RNA than at the protein level. This is unexpected for a transcription factor binding to the promoter region of ON (Wan *et al*, 2001; Roy and Sen, 2005). The findings are, however, in line with an inhibition of translation, as observed for miRNAs. Whether or not this consideration is valid should be investigated in future experiments.

Currently, there are contradicting assumptions on the role of ON in cancer development, including PDAC (Podhajcer *et al*, 2008). Osteonectin has been found increased in malignant tumours and was described to correlate in intestinal-type gastric cancer with local tumour growth, nodal spread and tumour stage (Franke *et al*, 2009). Accordingly, ON was considered a promising novel target for cancer treatment by these authors.

However, up-regulation alone is not sufficient to establish an oncogenic effect, since it could also be interpreted as a defence mechanism. This view is supported by the following findings. In primary PDAC, ON was detected in tumour cells, their adjacent ECM as well as in fibroblasts and endothelial cells. In metastatic PDAC, the strongest ON expression was detected in the surrounding stroma, whereas it remained below detection level within the metastases. This correlates well with the observation that ON has an anti-proliferative effect *in vitro* (Guweidhi *et al*, 2005). In addition, ON knockout mice are distinctly more prone to enhanced growth of pancreatic tumours following both subcutaneous and orthotopic tumour cell implantation. Finally, the absence of ON in pancreatic cancer cells was reported to be due to hyper-methylation of the protein's promoter in 16 of 18 cell lines investigated (Sato *et al*, 2003). All these observations point to a role of ON, which suggests that it could be an anti-tumourigenic protein rather than a protein responsible for tumour progression (Zhivkova-Galunska *et al*, 2010). In line with this assumption, lack of ON expression in colorectal cancer was recently reported to be associated with poor prognosis (Yang *et al*, 2007). The direct relationship between HOXC8 and ON, in turn, is supportive of the assumption that HOXC8 participates in or regulates the anti-tumourigenic role of ON.

However, a classical tumour suppressor requires the continued presence of its gene product and this property lacks HOXC8, since in normal tissues, HOXC8 is expressed at basal levels only. This transcription factor is physiologically being up-regulated by signals emitted in response to changes that are not known so far. Based on the connection between HOXC8 and ON, it could be speculated that wound formation (including certain aspects of tumour formation) could be a trigger.

Another possibility could be that HOXC8 up-regulation is associated with tumour cell dissemination from the primary, a function that is dispensable at later, metastasised stages. A dual role of HOXC8 has been described in prostate carcinoma, with a repressive function on gene induction at androgen response element-regulated genes as well as a function promoting invasiveness of this tumour. This observation, however, does not seem to be paradigmatic for PDAC.

In conclusion, Hoxc8 mRNA expression in human pancreatic cancer cell lines was inversely related to their capability to grow in the liver of nude rats and successful down-regulation of HOXC8 expression caused increased proliferation, migration and colony formation in Suit2-007, Panc-1 and MIA PaCa-2 PDAC cells, respectively. The Hoxc8 mRNA levels in diseased human pancreas tissues were significantly increased over normal in PDAC, but negatively related to tumour stage (*P*=0.09). In primary and metastatic tumour samples, immunohistochemistry staining for HOXC8 was always stronger in surrounding than in neoplastic tissues. Furthermore, grading of primary carcinomas was negatively associated with the extent and intensity of HOXC8 staining. Liver metastases showed the lowest HOXC8 expression of all neoplastic lesions. These data indicate that HOXC8 expression is inversely related to PDAC progression and metastases and might thus serve as marker for PDAC progression.

## Figures and Tables

**Table 1:** Primers and siRNA oligomers used

**Gene**	**Primer sequences** a
*Hoxc8* human	5′-CAT GTT TCC ATG GAT GAG ACC-3′ forward 5′-TGA TAC CGG CTG TAA GTT TGC-3′ reverse
*OSTEONECTIN (ON, SPARC)* human	5′-CCC GCT TTT TCG AGA CCT-3′ forward 5′-CAA GAT CCT TGT CGA TAT CCT TC-3′ reverse
*OSTEOPONTIN (OPN, SPP1)* human	5′-CGC AGA CCT GAC ATC CAG T-3′ forward 5′-GGC TGT CCC AAT CAG AAG G-3′ reverse
*GAPDH* human	5′-AGC CAC ATC GCT CAG ACA-3′ forward 5′-GCC CAA TAC GAC CAA ATC C-3′ reverse
siRNA	**siRNA sequences^b^**
siRNA *Hoxc8* negative control 1	5′-GAGUCCGAACCCUUAGUAUGCACUA-3′ 5′-UAGUGCAUACUAAGGGUUCGGACUC-3′
siRNA *Hoxc8*-1	5′-GAGACGCCUCCAAAUUCUAUGGCUA-3′ 5′-UAGCCAUAGAAUUUGGAGGCGUCUC-3′
siRNA *Hoxc8* negative control 2	5′-UGGUCAGCCAGAGAGACGAUAGGAA-3′ 5′-UUCCUAUCGUCUCUCUGGCUGACCA-3′
siRNA *Hoxc8*-2	5′-UGGGACUGACCGAGAGACAAGUGAA-3′ 5′-UUCACUUGUCUCUCGGUCAGUCCCA-3′

Abbreviation: SPARC=secreted protein acidic and rich in cysteine.

a Obtained from Dharmacon (Lafayette, CO, USA).

b Obtained from Invitrogen.

**Table 2:** mRNA expression of Hoxc8, OPN and ON in 15 pancreatic cancer cell lines and correlation with their ability to grow in the liver of nude rats

**Cell lines**	**Hoxc8** a	**OPN** b	**ON** c	**Growth in the liver of nude rats**
DANG	++++	−	+++	−
Panc 1	++++	−	++	−
Suit2-007	++++	++	+	+++
A8 18-4	+++	−	−	−
MIA PaCa-2	++	−	+	−
Capan-1	++	−	+	−
PATU 390	++	−	−	−
SU 8686	+	++	++	−
CFPAC 1	−	+	+	+
AsPC1	−	++	−	++
Panc 89	−	+	−	+
Colo 357	−	++	−	++
ASML	−	+	+	+++
Suit2-013	−	+++	−	++
BxPC3	−	++	−	+

Abbreviations: ON=osteonectin; OPN=osteopontin.

a Hoxc8: copies per *μ*l from qRT–PCR. –: mRNA expression below detection level or barely detectable (0–20 copies per *μ*l); +: 20–50 copies per *μ*l; ++: 51–200 copies per *μ*l; +++: 201–500 copies per *μ*l; ++++: >500 copies per *μ*l.

b OPN: peak area (arbitrary units) from capillary electrophoresis. –: mRNA expression below detection level; +: 0–3; ++: >3–6; +++: > 6.

c ON: peak area (arbitrary units) from capillary electrophoresis. –: mRNA expression below detection level or barely detectable (0.0–0.5); +: >0.5–1; ++: >1–2; +++: >2.
