# Supplementary material for: Expression of HOXC8 is inversely related to the progression and metastasis of pancreatic ductal adenocarcinoma
Source: Br J Cancer. 2011 Jun 28;105(2):288–95. doi: 10.1038/bjc.2011.217 (PMC3142801; doi:10.1038/bjc.2011.217)
Supplement: Supplementary information [file bjc2011217x1.doc]

**Supplementary table: Overview of pancreatic cancer cell lines used**

| Cell line/ Tissue | Origin | Source | Growth conditions ^d)^ |
| --- | --- | --- | --- |
| A818-4  ^a)^  ascites | Ascites PDAC | Obtained from Clinic for Surgery, University of Heidelberg | RPMI 1640 + 10% FBS |
| AsPC-1 ^a)^  ascites | Established from nude mouse xenografts initiated with cells from the PDAC ascites of a 62-year-old female Caucasian | ATCC® Number: **CRL-1682™** | RPMI 1640 + 10% FBS |
| BxPC-3 ^a)^  prim. PDAC | Established from a primary PDAC of a 61-year-old female patient | ATCC® Number:  **CRL-1687™** | RPMI 1640 + 10% FBS |
| COLO 357 ^a)^ lymph node metastasis | Established from a PDAC lymph node metastasis of a 77- year- old female | Obtained from Clinic for Surgery, University of Heidelberg | RPMI 1640 + 10% FBS |
| DAN-G ^a)^  prim. PDAC | Established in 1985 from a primary PDAC | DSMZ no.:  ACC 249 | RPMI 1640 + 10% FBS |
| PANC 89^a)^ lymph node metastasis | Established from a PDAC lymph node metastasis | Obtained from Clinic for Surgery, University of Heidelberg | RPMI 1640 + 10% FBS |
| Patu 390 ^a)^  prim. PDAC | Established from primary PDAC | Obtained from Clinic for Surgery, University of Heidelberg | RPMI 1640 + 10% FBS |
| S2-007 ^a)^  liver metastasis | Subclone derived from Suit-2 cells, established from PDAC liver metastasis | Obtained from K. M. Debatin, Medical Faculty, University of Ulm | McCoy’s medium + 10% FBS |
| S2-013 ^a)^  liver metastasis | Subclone derived from Suit-2 cells, established from PDAC liver metastasis | Obtained from Clinic for Surgery, University of Heidelberg | McCoy’s medium + 10% FBS |
| SU.86.86 ^a)^  liver metasta­sis | Established from a wedge biopsy of PDAC liver metastasis of a 57-year-old female Caucasian | ATCC® Number:  **CRL-1837™** | RPMI 1640 + 10% FBS |
| AsML ^b), c)^ | In vivo strong metastatic but hardly invasive subclone of BSp 73 | Obtained from M. Zöller, DKFZ, Heidelberg | RPMI 1640 + 10% FBS |
| MIA PaCa-2 ^a)^  prim. PDAC | Established from an undifferentiated PDAC of a 65- year- old male Caucasian | ATCC® Number:  CRL-1420 | Dulbecco's Modified Eagle's Medium + 10% FBS + 2.5% horse serum |
| Panc-1 ^a)^  prim. PDAC | Established from the primary PDAC of a 56-year-old male Caucasian | ATCC® Number: **CRL-1469™** | Dulbecco's Modified Eagle's Medium + 10% FBS |
| Capan-1^a)^  liver metastasis | Established in 1974 from the PDAC liver metastasis of a 40-year-old male Caucasian | ATCC® Number:  **HTB-79™** | Iscove's Modified Dulbecco's Medium + 20% FBS |
| CFPAC-1 ^a)^ liver metastasis | Established from the PDAC liver metastasis of a 26-year-old male Caucasian with cystic fibrosis | ATCC® Number:  **CRL-1918™** | Iscove's Modified Dulbecco's Medium + 10% FBS |

^a)^ – Human origin ^b)^ – rat origin ^c)^ complete name of ASML: BSp 73 ASML
 ^d)^ All media are supplemented with 2mM L- glutamine

**Supplementary methods S1**

Two sequential 3 µm thick paraffin-embedded tissue sections were placed on the same slide, de-paraffinized, and re-hydrated in progressively decreasing concentrations of ethanol. One section was used for analysis while the other was used as negative con­trol. Antigen retrieval was performed by boiling the slides in 10 mM citrate buffer twice for 10 min. Peroxidase activity was quenched with a 3% H_2_O_2_ solution in 30% methanol at room temperature for 10 min. The slides were then washed in washing buffer (10 mM Tris-HCl, 0.85% NaCl, 0.1% bovine serum albumin, pH 7.4) and incu­bated with mouse monoclonal anti- HOXC8 antibody (Abnova) diluted 1:50 in a universal blocking reagent (DAKO Corporation, Carpentaria, CA). After an overnight incubation at 4°C, slides were washed with Tris buffer supplemented with 0.05% Tween-20 (TBS-T) and exposed to the HRPO-linked anti-mouse secondary antibody (Amersham Interna­tional, Bucking­hamshire, UK) for 45 min at room temperature. Colour reaction was car­ried out by incu­bation for 4 min with liquid AB+ substrate and counterstaining by Mayer's hematoxylin solution. The results were evaluated by a pathologist (FB) for lesion sever­ity, intensity of staining, and percentage of positive cells after inspection of all fields in the tissue sec­tions. For evaluation of the ductal structures, sections were scanned at low (
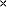
 50) magnifi­cation. Each duct identified was then evaluated at higher magnifica­tions (
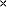
 200/
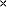
400). Any duct with staining present in more than 10% of the epithelial cells was counted as positive. When more than 30% of the ducts in a section were posi­tive, that section was counted as positive. Cancer sections without the formation of duct-like structures were scored as positive if more than 10% of the cancer cells were stained. Analysis and semi-quantitative evaluation were performed using an Axioplan-2 microscope (Carl Zeiss, Jena, Germany).

**Supplementary methods S2**

Cells (control and transfected) were incubated, harvested and washed in PBS. Thereaf­ter they were counted (Neubauer chamber) and cell pellets (2x0^6^ cells) were suspended in 200 µl buffer [0.1 M NaCl, 0.01 M Tris. Cl (pH 7.6), 0.001 M EDTA (pH 8.0)] contain­ing protease inhibitor cocktail (Roche Diagnostics, Mannheim, Germany). After that the cells were lysed by adding 200µl of lysis buffer [100 mM TrisCl (pH 6.8), 200mM dithio­threitol (Serva, Heidelberg, Germany), 4% SDS, 0.2% bromophenol blue, and 20% glyc­erol]. After vigorously vortexing, lysates were boiled for 10 minutes in a water bath at 100°C and centrifuged at 14000 rpm for 10 min. at room temperature. The protein con­centration of the lysate was determined using the BCA protein assay from Pierce (Rockford, Illinois, USA) according to the manufacturer’s recommendation. Cell lysates corresponding to 150,000 cells were loaded onto a 4-12 % SDS polyacrylamide gel (In­vitrogen), separated by electrophoresis and transferred to a PVDF membrane (Roche Applied Science, Mannheim, Germany ), blocked with 5% skimmed milk in PBS and in­cubated with the respective first antibody [HOXC8: mouse - antihuman monoclonal anti­body (Abnova GmbH Heidelberg); OPN: rabbit –antihuman polyclonal or mouse - anti­human monoclonal antibody; ON: rabbit – antihuman polyclonal (from Immundiagnostik Comp. Bensheim, Germany) for 2h in PBS; containing 0.5% milk and 0.5% Triton X-100. After washing with PBS, containing 0.05% Tween 20, an HRP – conjugated anti-mouse, anti-rabbit or anti-chicken secondary antibody (all from Santa Cruz Biotechnology, Hei­delberg, Germany) and ECL (GE Healthcare, Freiburg, Germany) were used to detect the respective pro­teins by exposing the membrane to an X ray film (Kodak Biomax, Rochester, New York). To control for variations in loading, the membranes were stripped for 30 min. at 56°C in stripping solution (62.5 mM Tris HCl, (pH 6.8); 2% SDS; 0.007% β-mercapto­ethanol). There­after they were re-probed with an antibody against β-actin or ERK-2 (mouse monoclonal anti­body, Santa Cruz Biotechnology), and a sec­ondary goat anti mouse polyclonal antibody (Santa Cruz Biotechnol­ogy).

**Supplementary methods S3**

100µl RPMI 1640 medium per well containing 5×10^3^  Suit2-007, Panc-1 or MIA PaCa-2 cells were plated onto 96-well plates (Microtest™, Becton Dicktinson, Heidelberg, Ger­many). After 24h, the cells were exposed to the respective si-RNA and then further incu­bated for 48-96 h under standard cell culture conditions. The number of surviving cells was determined by adding 10lµl/well of 3-[4,5-dimethylthiazol-2-yl]-2,5 diphenyltetra­zolium bromide; (MTT; 10mg/ml) to each well. The plates were further incubated for 4 h and thereafter the supernatant was removed, and formazan crystals that had been de­veloped were dissolved by adding of 100µl acidified 2-propanol/well (0.04 N HCl). Ab­sorption was measured by an automated microtiter plate reader (Anthos Mikrosysteme, Krefeld, Germany) at 540 nm, reference filter 690 nm.
